# Entanglement of propagating optical modes via a mechanical interface

**DOI:** 10.1038/s41467-020-14768-1

**Published:** 2020-02-18

**Authors:** Junxin Chen, Massimiliano Rossi, David Mason, Albert Schliesser

**Affiliations:** 1 Niels Bohr Institute, University of Copenhagen, 2100 Copenhagen, Denmark; 2 Center for Hybrid Quantum Networks (Hy-Q), Niels Bohr Institute, University of Copenhagen, 2100 Copenhagen, Denmark; 3 Present Address: Department of Applied Physics, Yale University, New Haven, CT USA

**Keywords:** Optical physics, Optomechanics, Quantum information, Quantum mechanics

## Abstract

Many applications of quantum information processing (QIP) require distribution of quantum states in networks, both within and between distant nodes. Optical quantum states are uniquely suited for this purpose, as they propagate with ultralow attenuation and are resilient to ubiquitous thermal noise. Mechanical systems are then envisioned as versatile interfaces between photons and a variety of solid-state QIP platforms. Here, we demonstrate a key step towards this vision, and generate entanglement between two propagating optical modes, by coupling them to the same, cryogenic mechanical system. The entanglement persists at room temperature, where we verify the inseparability of the bipartite state and fully characterize its logarithmic negativity by homodyne tomography. We detect, without any corrections, correlations corresponding to a logarithmic negativity of *E*_N_ = 0.35. Combined with quantum interfaces between mechanical systems and solid-state qubit processors, this paves the way for mechanical systems enabling long-distance quantum information networking over optical fiber networks.

## Abstract (web summary)

Applications of quantum information processing require distribution of quantum states for linking nodes in networks and mechanical oscillators can create versatile links. Here, the authors describe continuous variable entanglement between two optical modes mediated by a mechanical oscillator.

## Introduction

Entanglement is a crucial resource for quantum information processing (QIP)^1^. As such, the ability to entangle fields of arbitrary wavelength will be important for linking nodes in heterogeneous QIP networks^2^. Mechanical oscillators are uniquely poised in their ability to create such links, thanks to the frequency-independence of the radiation pressure interaction^3,4^ and their ability to couple to various solid-state qubits^5–10^. The ability to entangle two radiation fields via a common mechanical interaction was outlined 20 years ago^11–13^, and the intervening decades have seen the development of optomechanical devices^14^ which are robustly quantum mechanical and routinely integrated into hybrid systems.

**Fig. 1:**
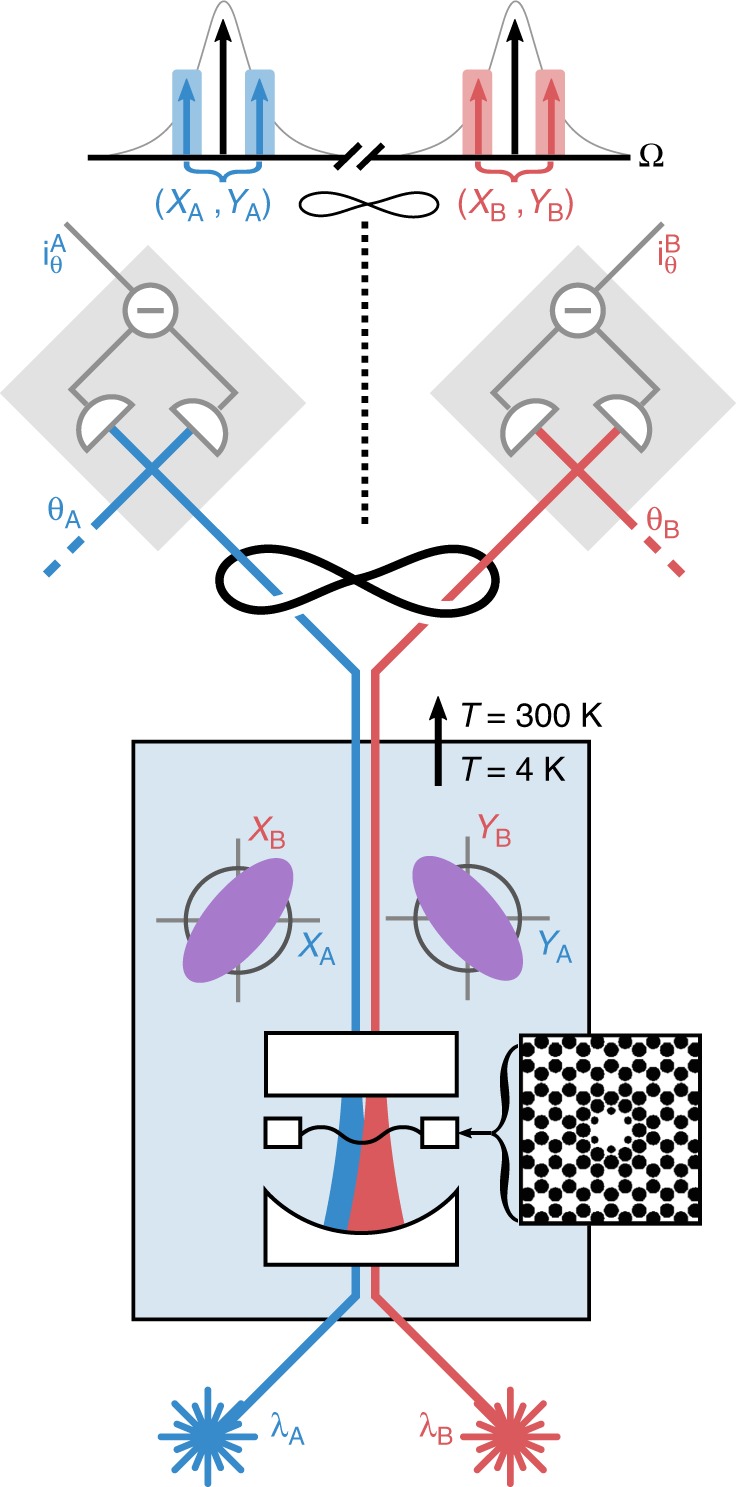
Experimental setup. Two lasers (red and blue) simultaneously drive an optomechanical cavity, kept in a helium flow cryostat. The inset shows the structure of the soft-clamped mechanical resonator (Si_3_N_4_ in white, holes in black). Exiting the cavity, the optical fields possess nonlocal correlations, illustrated by the squeezed phase space ellipses. After the cavity, the two lasers are physically separated and detected simultaneously by balanced homodyne detectors, with local oscillators locked at phases *θ*_*A*_, *θ*_*B*_. The top of the figure shows a frequency diagram of the relevant optical modes. The two cavity drives are shown in black, with scattered mechanical sidebands of laser *A* and *B* shown in blue and red, respectively. The sideband quadrature modes considered in the paper correspond to combinations of both scattered sidebands, as indicated by the blue and red shaded areas.

Recently, mechanically mediated entanglement has been reported between propagating microwave fields^15^ as well as two superconducting qubits^16^. In both cases, the entanglement remained confined to the dilution refrigerator in which it was created. Here, we create mechanically entangled optical fields for the first time, spanning up to 100 nm in wavelength, by utilizing an extremely coherent mechanical platform (Fig. 1). Moreoever, while the entangling mechanical interface resides at cryogenic temperatures, it is compatible with highly efficient light extraction and collection, such that we can directly measure the entanglement at room temperature, without noise subtraction or other indirect inference. This in turn means that the entangled optical fields could easily be distributed for QIP applications.

## Results

### Theoretical model

We consider two propagating optical fields (labeled by *j* = *A*, *B*), from which one can identify a pair of temporal modes with quadratures ${\hat{X}}_{j},{\hat{Y}}_{j}$. We take the variance of these modes to be 1/2 in their ground state. From these modes, one can construct joint Einstein–Podolsky–Rosen (EPR)-type variables ${\hat{X}_{\pm }}={\hat{X}_{A}}\pm {\hat{X}_{B}}$ and ${\hat{Y}_{\pm }}={\hat{Y}_{A}}\pm {\hat{Y}_{B}}$, which form the basis for various entanglement criteria^17,18^. We adopt the common Duan–Giedke–Cirac–Zoller (DGCZ) criterion^17^ for the inseparability ${\mathcal{I}}$, which states that the two modes are inseparable if their variances (*V*) satisfy

1 $${\mathcal{I}}\equiv \frac{V({\hat{X}}_{+})+V({\hat{Y}}_{-})}{2} \, < \, 1.$$

To further quantify this entanglement, one can utilize the system’s covariance matrix, *σ*, which fully characterizes the correlations between various quadratures. From this matrix, it is straightforward to calculate the symplectic eigenvalues of its partial transpose, ${\tilde{\nu }_{\pm }}\!$^19^. These eigenvalues offer a condition for separability ($2{\tilde{\nu }}_{-}\ge 1$), as well as a tool to calculate a common measure of entanglement, the logarithmic negativity ${E}_{N}=\max \left[0,-{\mathrm{log}\,}_{2}\ 2{\tilde{\nu }}_{-}\right]$. We note that 2${\tilde{\nu }}_{-}$ also corresponds to the minimum value of ${\mathcal{I}}$ possible when optimizing over local operations on either subsystem (e.g., squeezing and rotation)^20^. Thus, 2${\tilde{\nu }}_{-}$ serves as a lower bound for any DGCZ measurement.

In an optomechanical setting, in which a mechanical resonator (unitless position $\hat{q}$, momentum $\hat{p}$) is linearly coupled to two independent, resonantly driven cavity modes (amplitude ${\hat{X}}_{j}^{{\rm{cav}}}$, phase ${\hat{Y}}_{j}^{{\rm{cav}}}$, *j* = *A*, *B*), the interaction Hamiltonian can be written as: ${\hat{H}}_{{\rm{int}}}=-{\sum }_{j}2\hslash {g}_{j}{\hat{X}}_{j}^{{\rm{cav}}}\hat{q}$, where *ℏ* is the reduced Planck constant, and *g*_*j*_ are the field-enhanced optomechanical coupling rates. We consider an unresolved-sideband system, in which the cavity decay rates, *κ*_*j*_ are much faster than the mechanical frequency Ω_m_ and mechanical energy dissipation rate Γ_*m*_. We also assume that the two cavity modes are driven symmetrically by coherent states, such that their induced quantum backaction rates, ${\Gamma }_{j}^{{\rm{qba}}}=4{g}_{j}^{2}/{\kappa }_{j}$ are equal: ${\Gamma }_{A}^{{\rm{qba}}}={\Gamma }_{B}^{{\rm{qba}}}\equiv {\Gamma }_{{\rm{qba}}}$. The quadratic interaction preserves the Gaussianity of the state. The following equations of motion link the input and output optical fields

2a $${\hat{X}}_{j}^{{\rm{out}}}(t)=-{\hat{X}}_{j}^{{\rm{in}}}(t),$$

2b $${\hat{Y}}_{j}^{{\rm{out}}}(t)= 	-{\hat{Y}}_{j}^{{\rm{in}}}(t)-2\sqrt{{\Gamma }_{{\rm{qba}}}}{\chi }_{{m}}(t)* [\sqrt{2{\Gamma }_{m}}{\hat{P}}_{{\rm{in}}}(t) \\ 	+{\sum _{i = A,B}}2\sqrt{{\Gamma }_{{\rm{qba}}}}{\hat{X}}_{i}^{{\rm{in}}}(t)],$$

where ${\hat{X}}_{j}^{{\rm{in}}}$ and ${\hat{Y}}_{j}^{{\rm{in}}}$ are input vacuum noise quadratures, *χ*_*m*_ is the mechanical susceptibility, Γ_*m*_ is the mechanical energy dissipation rate, * indicates convolution, and ${\hat{P}}_{{\rm{in}}}$ is the mechanical thermal noise operator.

In Eq. (2), we see that the quantum amplitude fluctuations of each laser drive the mechanical system, whose motion is then imprinted on the optical phase. This is the same mechanism that drives ponderomotive squeezing of a single laser^21^, but in this case there are also cross-correlations between the lasers. More insight can be had by moving to the joint mode basis (see Supplementary Note 2), where one finds that the system decouples into a sum mode undergoing ponderomotive squeezing, and a difference mode which remains dark to all mechanical dynamics. It is this squeezing of a joint (nonlocal) mode which results in the “ponderomotive entanglement” we study here. We note that Eq. (2) also closely mirror those describing four-mode squeezing based on the Kerr nonlinearity in glass^22^, in which the response function *χ*_*m*_ is effectively instantaneous. Both approaches are members of a broader class of settings that enable, in principle, quantum-non-demolition measurements of light^23,24^.

Homodyne detection allows measurement of optical quadratures in a rotated basis defined by the local oscillator phase, *θ*_*j*_. Filtering the homodyne signal at frequency Ω with a mode function *h*(*t*) yields the sideband quadratures of a particular temporal mode at time *t*:

3a $${\hat{X}}_{j}^{{\theta }_{j}}(t)={\int _{-\infty }^{t}}ds\ \cos (\Omega s)h(t-s)\left({\hat{X}}_{j}^{{\rm{out}}}(s)\cos ({\theta }_{j})+{\hat{Y}}_{j}^{{\rm{out}}}(s)\sin ({\theta }_{j})\right),$$

3b $${\hat{Y}}_{j}^{{\theta }_{j}}(t)={\hat{X}}_{j}^{{\theta }_{j}+\pi /2}(t),$$

where *θ*_*j*_ is the homodyne angle. Note that the quadratures ${\hat{X}}_{j}^{{\theta }_{j}}$ available in a homodyne detector contain a pair of sidebands, symmetric to the carrier, as illustrated at the top of Fig. 1. As there are in total four optical modes involved, correlations between such modes are sometimes called four-mode-squeezing (see Supplementary Note 3), in contrast to the entangled microwave modes recently analyzed in a heterodyne scheme^15^. In the following model, we consider the limit of long filter times, in which *h* effectively selects a single Fourier component^20^. Furthermore, since the system is stationary, we drop the time argument *t* and focus on the ensemble statistics of these modes.

Within this model, one can obtain a simple expression for the DGCZ inseparability criterion (see Supplementary Note 1 for detail)

4 $${{\mathcal{I}}}_{\Theta ,\Omega }^{{\rm{ideal}}}=	 \,\,1+8{\Gamma }_{{\rm{qba}}}| {\chi }_{{m}}(\Omega ){| }^{2}{\Gamma }_{{\rm{dec}}}\left(1+\cos (2\Theta )\right)\\ 	-4{\Gamma }_{{\rm{qba}}}{\rm{Re}}\left[{\chi }_{{m}}(\Omega )\right]\sin (2\Theta ),$$

where Θ ≡ (*θ*_*A*_ + *θ*_*B*_)/2. The first term is the contribution from shot noise at the detectors. The second term is the contribution from mechanical motion, where the total decoherence rate ${\Gamma }_{{\rm{dec}}}=2{\Gamma }_{{\rm{qba}}}+{\Gamma }_{m}({\bar{n}}_{{\rm{th}}}+1/2)$ includes both quantum backaction sources and thermal motion. The third term corresponds to correlation between two beams, again in close analogy to ponderomotive squeezing^21^. In practice, there is always optical loss, which admits vacuum noise that degrades the detected correlations. This is described by a collection efficiency *η*_*A*_ = *η*_*B*_ ≡ *η* < 1, with which the inseparability of the detected optical states becomes ${{\mathcal{I}}}_{\Theta ,\Omega }=\eta {{\mathcal{I}}}_{\Theta ,\Omega }^{{\rm{ideal}}}+(1-\eta )$. By defining a combined measurement efficiency *η*_meas_ = 2*ηΓ*_qba_/*Γ*_dec_, one can show (see Supplementary Note 1) that the minimum of ${{\mathcal{I}}}_{\Theta ,\Omega }$ is given by 1 − *η*_meas_/2. By further calculating the full covariance matrix for this toy model (see Supplementary Note 1), one can show that $\min \{2{\tilde{\nu }}_{-}\}=\sqrt{1-{\eta }_{{\rm{meas}}}}$, that is, the system can generate arbitrarily strong entanglement as *η*_meas_ → 1.

### Experimental setup

In practice, the optical fields become entangled via their shared interaction with a 3.6 mm × 3.6 mm × 20 nm soft-clamped Si_3_N_4_ membrane^25^. The vibrational mode of central defect has a frequency of Ω_*m*_ = 2*π* × 1.139 MHz, and a quality factor *Q* = 1.04 × 10^9^ at a temperature of 10 K, which corresponds to a mechanical linewidth of Γ_*m*_ = 2*π* × 1.10 mHz.

As illustrated in Fig. 1, the membrane is inserted in the middle of an optical cavity^26,27^, addressed by two lasers with wavelength  ~796 nm. These lasers are orthogonally polarized and populate the cavity in two different longitudinal modes separated by  ~0.3 THz, with linewidths of *κ*_*A*_ = 2*π* × 13.3 MHz and *κ*_*B*_ = 2*π* × 12.6 MHz. With this setup, we achieve ${\Gamma }_{A}^{{\rm{qba}}}\approx 2\pi \times 1.35$ kHz and ${\Gamma }_{B}^{{\rm{qba}}}\approx 2\pi \times 0.89$ kHz, which easily exceed the thermal decoherence rate ${\Gamma }_{{\rm{m}}}{\bar{n}}_{{\rm{th}}}\approx 2\pi \times 0.20$ kHz. We measure the optical quadratures of the cavity output fields using two separate balanced homodyne detectors, achieving overall collection efficiencies of *η*_*A*_ = 60% and *η*_*B*_ = 77%. This gives a combined measurement efficiency of *η*_meas_ = 58% (see Supplementary Note 5).

By combining slope and dither lock techniques we are able to arbitrarily stabilize the phase of the local oscillators in the range [0, 2*π*). The photocurrent of each balanced homodyne detector is digitized with a 15 MSa/s analog-to-digital converter. We numerically demodulate the photocurrents at frequency Ω/(2*π*) = 1.1416 MHz, and low-pass filter the result with bandwidth 200 Hz. (This bandwidth is narrow compared to the mechanical feature of interest, allowing us to apply the infinitely long-filter limit of the model).

### Inseparability

**Fig. 2:**
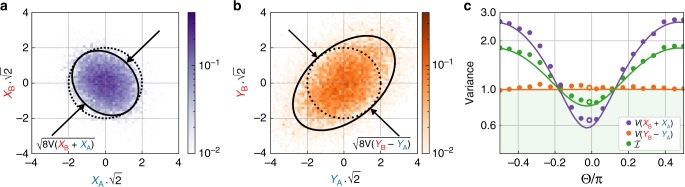
EPR quadrature statistics. **a**, **b** 2D histograms of raw *X* and *Y* quadrature data, respectively, for Θ ≈ 0. The black dashed circle indicates (2×) the vacuum noise variance, which has a radius of $1/\sqrt{2}$ (note the axes' scale factor of $\sqrt{2}$). The solid black ellipses indicate (2×) the covariance ellipse of the measured data. The black arrows indicate the diagonal/anti-diagonal variances which are relevant for calculating the DGCZ criterion. **c** Homodyne angle dependence of joint quadrature variances. The purple (orange) dots are the sum (difference) quadrature ${\hat{X}}_{+}$ (${\hat{Y}}_{-}$). The average of these yields the DGCZ inseparability criterion (green points). The measurement ensembles contain  ~10^4^ samples, such that the statistical standard error of the variance estimators is  ~2% of the reported values. This contains both the error in the estimation of the EPR variances and the error in the estimations of the vacuum noise variance. The solid line is the theoretical prediction.

We now proceed to characterize the variance of EPR-type variables, as introduced above, to compare with the DGCZ criterion. We choose a common basis *θ*_*A*_ ≈ *θ*_*B*_ ≈ Θ ≈ 0 and measure, in sequence, the combinations $\{{\hat{X}}_{A}^{\Theta },{\hat{X}}_{B}^{\Theta }\}$, $\{{\hat{Y}}_{A}^{\Theta },{\hat{Y}}_{B}^{\Theta }\}$, and vacuum noise (by blocking the cavity output). Figure 2a, b shows histograms of the measured quadrature data for the *X* and *Y* quadratures, along with reference lines for vacuum noise variance in black. Recalling the joint quadrature definitions, we note that the DGCZ criterion involves the diagonal and anti-diagonal variances of the *X* and *Y* histograms, respectively. In the figure, we clearly see squeezing in the former, and near-vacuum variance in the latter—thus already indicating violation of the DGCZ criterion. Quantitatively, we find ${\mathcal{I}}=0.83\pm 2 \% ({\rm{stat}}.)\pm 0.3 \% ({\rm{syst}}.)$. The statistical error comes from the number of samples used to estimate the EPR variances and the vacuum noise. The systematic error arises from the estimations of the vacuum noise variance, due to residual classical amplitude noise and mismatch in the photodiode responsivities, at the balanced homodyne detectors (see Supplementary Note 6). We also notice that the variances in the orthogonal directions are at the vacuum level. This does not violate the Heisenberg uncertainty relation, since the pairs of quadratures {${\hat{X}}_{A},{\hat{X}}_{B}$} and {${\hat{Y}}_{A},{\hat{Y}}_{B}$} commute with each other and are not canonically conjugate observables. We repeat such measurement for different homodyne angles (Θ ∈ [−*π*/2, *π*/2]) as shown in Fig. 2c. The solid lines are theoretical predictions based on a full model of optomechanical dynamics (taking, in particular, dynamical backaction^14^ into account, see Supplementary Note 1), using system parameters extracted from fits (see Supplementary Note 5). We find good agreement over all phases, firmly establishing that the two optical modes satisfy the DGCZ inseparability criterion. From Fig. 2c, we also notice that the best two-mode squeezing we achieve is, for the quadrature ${\hat{X}}_{+}$, 1.8 dB below the vacuum noise limit.

### Covariance matrix tomography

**Fig. 3:**
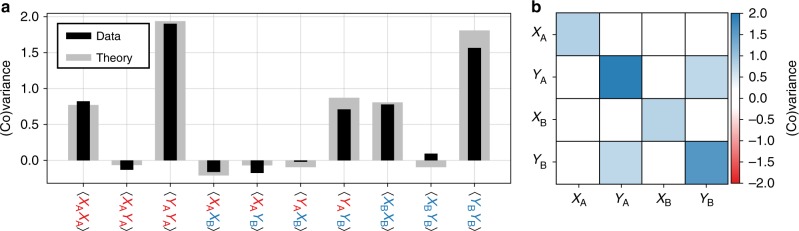
Covariance matrix of the two optical modes. **a** Measured (black) and predicted (gray) entries of the covariance matrix. The measurement ensembles contain  ~10^4^ samples, such that the standard error of the variance estimators is  ~2% of the reported values. **b** Matrix representation of the measured data, to highlight the location of the significant nonzero entries.

Having established entanglement, we now quantify it by reconstructing the covariance matrix by Gaussian homodyne tomography. By measuring five different pairs of angles {*θ*_*A*_, *θ*_*B*_} = {0, 0}, {*π*/2, *π*/2}, {0, *π*/2}, {*π*/2, 0}, {*π*/4, *π*/4}, we obtain all necessary intrasystem and intersystem correlations. The reconstructed covariance matrix and theoretical prediction are shown in Fig. 3. From this experimental data, we find a minimum symplectic eigenvalue $2{\tilde{\nu }}_{-}=0.79$, corresponding to *E*_*N*_ = 0.35.

### Frequency-dependent entanglement

**Fig. 4:**
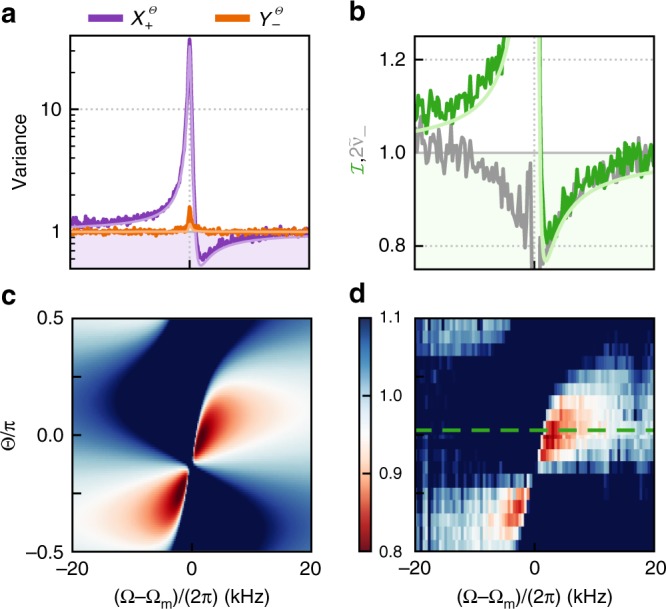
Frequency-dependent entanglement. **a** Variance of the EPR-type joint quadratures, ${\hat{X}}_{+}^{\Theta }$ (purple) and ${\hat{Y}}_{-}^{\Theta }$ (orange), from sum and difference of the two measured homodyne photocurrents, at angle Θ ≈ 0 and **b** derived inseparability (green), as a function of center frequency *Ω*. The solid lines in **a** and **b** are fit to a full model (see Supplementary Note 1). The dark gray line represents the minimum symplectic eigenvalue $2{\tilde{\nu }}_{-}$, obtained by optical homodyne tomography. The modes of the two laser fields are entangled whenever $2{\tilde{\nu }}_{-} \, < \, 1$. **c** Theory and **d** measurements of the inseparability ${\mathcal{I}}(\Theta ,\Omega )$. The green dashed line indicates the measurement shown in **b**. The horizontal axes are referenced to the bare mechanical frequency Ω_*m*_ = 2*π* × 1.139 MHz.

The previous results refer to a sideband quadrature mode at a particular frequency, Ω. We now examine how this entanglement varies as we sweep Ω near the mechanical resonance, Ω_*m*_.

(Note that for computational convenience, we do this by calculating noise spectral densities via the Fast Fourier Transform of the raw photocurrents, which corresponds to a mode function $h(t)=\theta (t)\theta (T-t)/\sqrt{T}$, where *θ*(*t*) is the Heaviside function and *T* ≈ 9 ms is the acquisition time). Figure 4 shows such a frequency-dependent inseparability, as well as its dependence on the homodyne measurement basis, Θ.

We see that the entanglement criteria can be met for frequencies above and below mechanical resonance, in a manner consistent with the dispersive third term in Eq. (4). The solid lines in Fig. 4a, b are theoretical predictions from the full model, based on a single set of system parameters, obtained from independent measurements or fits to account for drifts (see Supplementary Note 5). Moreover, similar to the measurement in Fig. 3, we can reconstruct the covariance matrix (and corresponding ${\tilde{\nu }}_{-}$) for each frequency bin, as shown in Fig. 4b. We see that, as expected, $2{\tilde{\nu }}_{-}$ serves as a lower bound for the inseparability ${\mathcal{I}}$. Since this bipartite Gaussian state is approximately symmetric, from $2{\tilde{\nu }}_{-}$ we can calculate the entanglement of formation, which is accepted as a proper measure of quantum correlations as a resource^15,28,29^. Integrating this quantity over a 30 kHz bandwidth yields an entanglement distribution rate of 753 ebits/s.

**Fig. 5:**
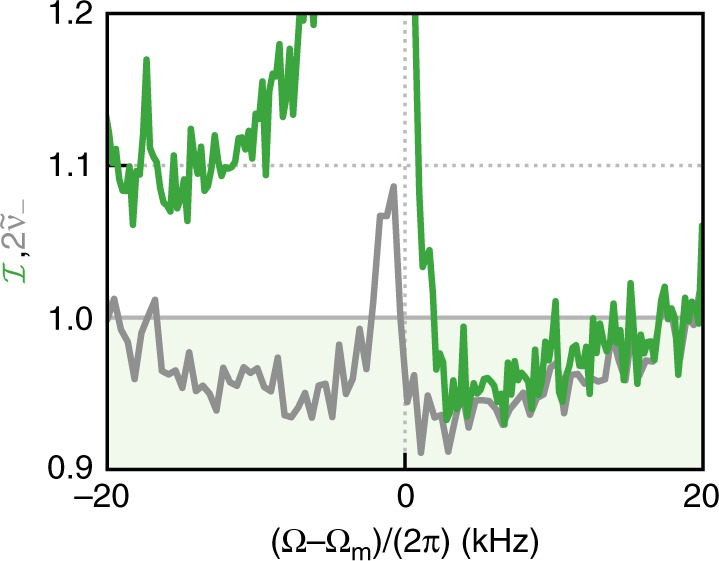
Entanglement spanning 120 nm in wavelength. Inseparability ${\mathcal{I}}(\Theta \approx 0,\Omega )$ (green) and minimum of the symplectic eigenvalue $2{\tilde{\nu }}_{-}$ (gray) from homodyne tomography. The horizontal axis is referenced to the bare mechanical frequency Ω_*m*_ = 2*π* × 1.139 MHz.

We emphasize that the optomechanical interaction which generated the entanglement presented above is fundamentally wavelength independent. To illustrate this, we move laser A to ~912 nm, and repeat the measurements of Fig. 4. As shown in Fig. 5, we observe a DGCZ variance below unity and a minimum symplectic eigenvalue $2{\tilde{\nu }}_{-}=0.92$ for a mode centered at *Ω* = 2*π* × 1.142 MHz with bandwidth 915 Hz. The performance is degraded compared to the previous results, due to less efficient light collection at  ~912 nm. Nevertheless, these results establish entanglement of two lasers separated by more than 100 nm in wavelength.

## Discussion

In conclusion, we have demonstrated quadrature entanglement of two nondegenerate optical beams via their common radiation–pressure interaction with a mechanical resonator. While applications in optical or microwave quantum communication are conceivable (as realized with entangled light sources based on more traditional optical or microwave parametric oscillators^28,30–33^), mechanical platforms offer unique possibilities. In particular, the combination of mechanically mediated microwave^15^ and optical (this work) entanglement could enable microwave-optical entanglement, based on membrane electro-opto-mechanical systems^34,35^. This would constitute a much-needed resource for networks of quantum computers based on superconducting qubits. In this context, it is noteworthy that the mechanical interface can in principle also store quantum information. Indeed, for the device presented here, the memory time is ca. 1 ms even for 10 K operation^27^, easily exceeding storage time in optical fibers.

In our work, entanglement is preserved from the cryogenic mechanical mediator all the way to the laser beams analyzed in room-temperature homodyne detectors. This enhances the prospects of a general class of hybrid quantum systems^3,4^ based on mechanical interfaces, which could harness entanglement between solid-state (e.g., spin or charge based) quantum systems, typically operating at low temperatures, and itinerant optical fields.

From a more fundamental perspective, it would be interesting to explore concepts at the interface of quantum measurement and entanglement. For instance, the optomechanical interaction in this work can also be interpreted as a strong quantum measurement of the mechanics. This system should be well-suited for studying the usually hard-to-access system-meter entanglement^36–38^.

## Supplementary information

Supplementary Information

## Data Availability

Source and raw data for Figs. 2–5 are available in the UCPH ERDA repository. The remaining data are available from the corresponding author upon request. The responsitory 10.17894/ucph.5351d84b-2f06-4157-b001-6dc17e75cb23.

## Code availability

The code used in the analysis of the data is available from the corresponding author upon reasonable request.

## References

[1] Horodecki R, Horodecki P, Horodecki M, Horodecki K (2009). Quantum entanglement. Rev. Mod. Phys..

[2] Kimble HJ (2008). The quantum internet. Nature.

[3] Stannigel K, Rabl P, Sørensen AS, Zoller P, Lukin MD (2010). Optomechanical transducers for long-distance quantum communication. Phys. Rev. Lett..

[4] Kurizki G (2015). Quantum technologies with hybrid systems. Proc. Natl Acad. Sci..

[5] Reed A (2017). Faithful conversion of propagating quantum information to mechanical motion. Nat. Phys..

[6] Pirkkalainen J-M (2013). Hybrid circuit cavity quantum electrodynamics with a micromechanical resonator. Nature.

[7] Satzinger KJ (2018). Quantum control of surface acoustic-wave phonons. Nature.

[8] Chu Y (2018). Creation and control of multi-phonon fock states in a bulk acoustic-wave resonator. Nature.

[9] Rabl P (2010). A quantum spin transducer based on nanoelectromechanical resonator arrays. Nat. Phys..

[10] Kolkowitz S (2012). Coherent sensing of a mechanical resonator with a single-spin qubit. Science.

[11] Giovannetti V, Mancini S, Tombesi P (2001). Radiation pressure induced Einstein-Podolsky-Rosen paradox. Europhys. Lett..

[12] Giannini S, Mancini S, Tombesi P (2003). Information theoretic aspects in ponderomotive systems. Quantum Inf. Comput..

[13] Manninen J, Asjad M, Ojajärvi R, Kuusela P, Massel F (2018). Clauser-Horne-Shimony-Holt Bell inequality test in an optomechanical device. Phys. Rev. A.

[14] Aspelmeyer M, Kippenberg TJ, Marquardt F (2014). Cavity optomechanics. Rev. Mod. Phys..

[15] Barzanjeh S (2019). Stationary entangled radiation from micromechanical motion. Nature.

[16] Bienfait A (2019). Phonon-mediated quantum state transfer and remote qubit entanglement. Science.

[17] Duan L-M, Giedke G, Cirac JI, Zoller P (2000). Inseparability criterion for continuous variable systems. Phys. Rev. Lett..

[18] Giovannetti V, Mancini S, Vitali D, Tombesi P (2003). Characterizing the entanglement of bipartite quantum systems. Phys. Rev. A.

[19] Adesso G, Serafini A, Illuminati F (2004). Extremal entanglement and mixedness in continuous variable systems. Phys. Rev. A.

[20] Zippilli S, Di Giuseppe G, Vitali D (2015). Entanglement and squeezing of continuous-wave stationary light. N. J. Phys..

[21] Safavi-Naeini AH (2013). Squeezed light from a silicon micromechanical resonator. Nature.

[22] Levenson M, Shelby R (1987). Four-mode squeezing and applications. J. Mod. Opt..

[23] Grangier P, Levenson JA, Poizat J-P (1998). Quantum non-demolition measurements in optics. Nature.

[24] Pontin A (2018). Quantum nondemolition measurement of optical field fluctuations by optomechanical interaction. Phys. Rev. A.

[25] Tsaturyan Y, Barg A, Polzik ES, Schliesser A (2017). Ultracoherent nanomechanical resonators via soft clamping and dissipation dilution. Nat. Nanotechnol..

[26] Thompson JD (2008). Strong dispersive coupling of a high-finesse cavity to a micromechanical membrane. Nature.

[27] Rossi M, Mason D, Chen J, Tsaturyan Y, Schliesser A (2018). Measurement-based quantum control of mechanical motion. Nature.

[28] Flurin E, Roch N, Mallet F, Devoret MH, Huard B (2012). Generating entangled microwave radiation over two transmission lines. Phys. Rev. Lett..

[29] Tserkis S, Ralph TC (2017). Quantifying entanglement in two-mode Gaussian states. Phys. Rev. A.

[30] Ou Z, Pereira SF, Kimble H, Peng K (1992). Realization of the Einstein-Podolsky-Rosen paradox for continuous variables. Phys. Rev. Lett..

[31] Silberhorn C (2001). Generation of continuous variable Einstein-Podolsky-Rosen entanglement via the Kerr nonlinearity in an optical fiber. Phys. Rev. Lett..

[32] Villar A, Cruz L, Cassemiro K, Martinelli M, Nussenzveig P (2005). Generation of bright two-color continuous variable entanglement. Phys. Rev. Lett..

[33] Grosse NB (2008). Observation of entanglement between two light beams spanning an octave in optical frequency. Phys. Rev. Lett..

[34] Bagci T (2014). Optical detection of radio waves through a nanomechanical transducer. Nature.

[35] Higginbotham AP (2018). Harnessing electro-optic correlations in an efficient mechanical converter. Nat. Phys..

[36] Vitali D (2007). Optomechanical entanglement between a movable mirror and a cavity field. Phys. Rev. Lett..

[37] Krisnanda T, Zuppardo M, Paternostro M, Paterek T (2017). Revealing nonclassicality of inaccessible objects. Phys. Rev. Lett..

[38] Gut, C. et al. Stationary optomechanical entanglement between a mechanical oscillator and its measurement apparatus. Preprint at https://arxiv.org/abs/1912.01635 (2019).

