# Author Correction: Entanglement of propagating optical modes via a mechanical interface

**DOI:** 10.1038/s41467-020-18516-3

**Published:** 2020-09-07

**Authors:** Junxin Chen, Massimiliano Rossi, David Mason, Albert Schliesser

**Affiliations:** 1 Niels Bohr Institute, University of Copenhagen, 2100 Copenhagen, Denmark; 2 Center for Hybrid Quantum Networks (Hy-Q), Niels Bohr Institute, University of Copenhagen, 2100 Copenhagen, Denmark; 3 Present Address: Department of Applied Physics, Yale University, New Haven, CT USA

Correction to: *Nature Communications* 10.1038/s41467-020-14768-1, published online 18 February 2020.

In the original version of this Article, the Data availability statement contained a link to a repository with the incorrect source data files. The correct link is: 10.17894/ucph.3e60afa5-6377-4488-ab27-850f1e7e8615.

This has been corrected in both the PDF and HTML versions of the article.

