# Supplementary material for: Entanglement of propagating optical modes via a mechanical interface
Source: Nat Commun. 2020 Feb 18;11:943. doi: 10.1038/s41467-020-14768-1 (PMC7028980; doi:10.1038/s41467-020-14768-1)
Supplement: Supplementary file 1 — Supplementary Information [file 41467_2020_14768_MOESM1_ESM.pdf]

Supplementary Information for "Entanglement of Propagating Optical Modes via a Mechanical Interface"

Junxin Chen,[1,2,*] Massimiliano Rossi,[1,2,*] David Mason,[1,2,3,*] Albert Schliesser,[1,2,†]

[1]Niels Bohr institute, University of Copenhagen, 2100 Copenhagen, Denmark

[2]Center for Hybrid Quantum Networks (Hy-Q), Niels Bohr Institute, University of Copenhagen, 2100 Copenhagen, Denmark

[3] Present address: Department of Applied Physics, Yale University, New Haven, CT, USA

[*]these authors contributed equally to this work

[†]To whom correspondence should be addressed; E-mail: albert.schliesser@nbi.ku.dk

Supplementary Note 1: Theory

Here we present a theoretical analysis of the experiments described in the main text. In the first section, we derive a model which fully describes the dynamics of an optomechanical system composed of two optical modes and a single mechanical mode. From these equations we derive a simplified, toy model, useful for the understanding of the physics. For the reader interested only in the toy model, we point out that it can be obtained from the complete equations in the limit of resonant lasers ($\Delta_j = 0$), unresolved cavities ($\kappa_j \gg \Omega_\mathrm{m}$) and identical measurement rates ($\Gamma_A^\mathrm{meas} = \Gamma_B^\mathrm{meas} = \Gamma_\mathrm{meas}$). We finally move to describe how one can fully characterize the entanglement of the state from the covariance matrix.

Three-mode optomechanical system

We consider two cavity modes A and B at optical frequency ω_{c_j}, described by the ladder operator $\hat{A}_j^\mathrm{cav} = \alpha_j^\mathrm{cav} + \hat{a}_j^\mathrm{cav}$ ($j = A, B$), where $\hat{a}_j^\mathrm{cav}$ is an operator representing fluctuations around the coherent amplitude α_j^cav and satisfying canonical commutation relations $[\hat{a}_j^\mathrm{cav}(t), \hat{a}_i^{\mathrm{cav},\dagger}(t')] = \delta_{ji}\delta(t-t')$. In the following, we assume that the cavity field α_j^cav is real. The mechanical resonator mode is described by the dimensionless position $\hat{Q} = Q + \hat{q}$ and its momentum $\hat{p}$. The fluctuations around the mean displacement Q are described

by the operator $\hat{q}$, which also satisfies canonical commutation relations $[\hat{q}(t), \hat{p}(t')] = i\delta(t - t')$.

The optomechanical coupling is described by the rates g_{0j}. In order to effectively enhance such coupling rate, we displace the cavity field by means of coherent driving lasers $\hat{A}_j^{\mathrm{in,L}} = (\alpha_j^{\mathrm{in}} + \hat{a}_j^{\mathrm{in,L}})e^{-i(\omega_{\mathrm{L},j}t+\phi_j)}$. The phase ϕ_j is needed in order to be consistent with the convention adopted of real intracavity field. We move each cavity modes to a frame rotating at the lasers' frequency $\omega_{\mathrm{L},j}$. In the limit of large coherent optical field α_j, the interaction can be linearized and the Hamiltonian in this interaction picture reads

$$\hat{H}_{\mathrm{IP}} = \hbar\Omega_{\mathrm{m}}\frac{\hat{q}^2 + \hat{p}^2}{2} - \sum_j \hbar\Delta_j \hat{a}_j^{\mathrm{cav},\dagger}\hat{a}_j^{\mathrm{cav}} - \sum_j \sqrt{2}\hbar g_j(\hat{a}_j^{\mathrm{cav},\dagger} + \hat{a}_j^{\mathrm{cav}})\hat{q}, \tag{S1}$$

where $\Delta_j = \tilde{\Delta}_j + \sqrt{2}g_{0j}Q = \omega_{\mathrm{L},j} - \omega_{\mathrm{c}_j}$ is the detuning between the laser field and the cavity modes and $g_j = g_{0j}\alpha_j^{\mathrm{cav}}$ is the enhanced optomechanical coupling.

The cavity modes are coupled to a lossy environment through two ports, hereby indicated as L and R, at rates $\kappa_{j,L}$ and $\kappa_{j,R}$ respectively. The total damping rate is $\kappa_j = \kappa_{j,L} + \kappa_{j,R}$. The mechanical system is also coupled to an environment, which leads to a damping rate Γ_{m}. We describe these open dynamics via the quantum Langevin equations. We also move to the quadrature representations of the optical modes, defined as $\hat{X}_j = (\hat{a}_j^\dagger + \hat{a}_j)/\sqrt{2}$ and $\hat{Y}_j = i(\hat{a}_j^\dagger - \hat{a}_j)/\sqrt{2}$. The equations of motions are

$$\dot{\hat{X}}_j^{\mathrm{cav}} = -\frac{\kappa_j}{2}\hat{X}_j^{\mathrm{cav}} - \Delta_j\hat{Y}_j^{\mathrm{cav}} + \sqrt{\kappa_{j,L}}\hat{X}_{j,\phi_j}^{\mathrm{in,L}} + \sqrt{\kappa_{j,R}}\hat{X}_j^{\mathrm{in,R}}, \tag{S2a}$$

$$\dot{\hat{Y}}_j^{\mathrm{cav}} = -\frac{\kappa_j}{2}\hat{Y}_j^{\mathrm{cav}} + \Delta_j\hat{X}_j^{\mathrm{cav}} + 2g_j\hat{q} + \sqrt{\kappa_{j,L}}\hat{Y}_{j,\phi_j}^{\mathrm{in,L}} + \sqrt{\kappa_{j,R}}\hat{Y}_j^{\mathrm{in,R}}, \tag{S2b}$$

$$\ddot{\hat{q}} = -\Gamma_m\dot{\hat{q}} - \Omega_{\mathrm{m}}^2\hat{q} + \Omega_{\mathrm{m}}\left(\sum_j 2g_j\hat{X}_j^{\mathrm{cav}} + \sqrt{2\Gamma_m}\hat{F}_{\mathrm{th}}\right). \tag{S2c}$$

The cavity modes are driven by optical vacuum noise $(\hat{X}_{j,\phi_j}^{\mathrm{in,L}}, \hat{Y}_{j,\phi_j}^{\mathrm{in,L}})$ and $(\hat{X}_j^{\mathrm{in,R}}, \hat{Y}_j^{\mathrm{in,R}})$, where $\phi_j = \tan^{-1}(\frac{\Delta_j}{\kappa_j/2})$ is the rotation due to the cavity response. The only non-zero symmetrized correlations are of the form $\overline{\langle\hat{X}_i(t)\hat{X}_j(t')\rangle} = \overline{\langle\hat{Y}_i(t)\hat{Y}_j(t')\rangle} = \frac{1}{2}\delta_{ij}\delta(t - t')$, where an overlined quantity refers to its symmetrized version, i.e. $\overline{\langle\hat{X}_i(t)\hat{X}_j(t')\rangle} \equiv \Big(\langle\hat{X}_j(t)\hat{X}_i(t')\rangle + \langle\hat{X}_i(t')\hat{X}_j(t)\rangle\Big)/2$. The mechanical system is driven by a Brownian force $\hat{F}_{\mathrm{th}}$. In the regime of underdamped motion ($\Gamma_{\mathrm{m}} \ll \Omega_{\mathrm{m}}$) and high-temperature of the bath ($k_{\mathrm{B}}T \gg \hbar\Omega_{\mathrm{m}}$)[1], this noise becomes Markovian and its symmetrized correlations is $\overline{\langle\hat{F}_{\mathrm{th}}(t)\hat{F}_{\mathrm{th}}(t')\rangle} = (\bar{n}_{\mathrm{th}} + 1/2)\,\delta(t - t')$.

We can experimentally only measure a field which leaks out from the cavity. We describe it with input-output relations, according to which the output field of mode j through the port i is

$$\hat{A}_j^{\mathrm{out},i} = \hat{A}_j^{\mathrm{in},i} - \sqrt{\kappa_{j,i}}\hat{A}_j^{\mathrm{cav}}. \tag{S3}$$

The systems of Equation (S2) and (S3) can be promptly solved in the Fourier domain (for an operator $\hat{a}(t)$, we define its Fourier transform $\hat{a}(\Omega) \equiv \int_{-\infty}^{\infty} dt e^{i\Omega t}\hat{a}(t)$). Let's introduce the cavity quadratures'

susceptibilities

$$u_j(\Omega) = \frac{\kappa_j/2 - i\Omega}{\Delta_j^2 + (\kappa_j/2 - i\Omega)^2}, \tag{S4a}$$

$$v_j(\Omega) = \frac{-\Delta_j}{\Delta_j^2 + (\kappa_j/2 - i\Omega)^2} \tag{S4b}$$

and the effective mechanical susceptibility modified by the dynamical backaction of the two optical modes

$$\chi_{\text{eff}}^{-1}(\Omega) \quad = \chi_{\text{m}}^{-1}(\Omega) - \textstyle\sum_j 4 g_j^2 v_j(\Omega), \tag{S5}$$

where $\chi_{\text{m}}(\Omega) = \Omega_{\text{m}} \left(\Omega_{\text{m}}^2 - \Omega^2 - i\Gamma_{\text{m}}\Omega\right)^{-1}$ is the bare mechanical susceptibility.
The quadratures of the field leaking through the most lossy port of our setup (the right ones) are

$$\begin{aligned} \hat{X}_j^{\text{out},R}(\Omega) = &- \sqrt{\kappa_{j,R}\kappa_{j,L}} \left(u_j(\Omega)\hat{X}_{j,\phi_j}^{\text{in,L}} + v_j(\Omega)\hat{Y}_{j,\phi_j}^{\text{in,L}} \right) \\ &- \kappa_{j,R} \left((u_j(\Omega) - 1/\kappa_{j,R})\hat{X}_j^{\text{in,R}} + v_j(\Omega)\hat{Y}_j^{\text{in,R}} \right) - 2g_j\sqrt{\kappa_{j,R}} v_j(\Omega)\hat{q}(\Omega), \end{aligned} \tag{S6a}$$

$$\begin{aligned} \hat{Y}_j^{\text{out},R}(\Omega) = &- \sqrt{\kappa_{j,R}\kappa_{j,L}} \left(-v_j(\Omega)\hat{X}_{j,\phi_j}^{\text{in,L}} + u_j(\Omega)\hat{Y}_{j,\phi_j}^{\text{in,L}} \right) \\ &- \kappa_{j,R} \left(-v_j(\Omega)\hat{X}_j^{\text{in,R}} + (u_j(\Omega) - 1/\kappa_{j,R})\hat{Y}_j^{\text{in,R}} \right) - 2g_j\sqrt{\kappa_{j,R}} u_j(\Omega)\hat{q}(\Omega) \end{aligned} \tag{S6b}$$

For each mode, we employ a balanced homodyne detector to measure these quadratures. By varying the phase θ_j of the local oscillator, we can detect quadratures in a rotated basis:

$$\hat{X}_j^{\theta_j}(\Omega) \equiv \hat{X}_j^{\text{out},R}(\Omega)\cos(\theta_j) + \hat{Y}_j^{\text{out},R}(\Omega)\sin(\theta_j). \tag{S7}$$

In order to verify the presence of entangled quadratures pairs between the two modes at some frequency Ω, we employ a common DGCZ criterion. In the frequency domain, this requires calculating symmetrized power spectral densities (PSD) and cross PSD between the modes. These PSDs are generally expressed by

$$\overline{S}^{\text{out}}_{\hat{X}_j^{\theta_j}\hat{X}_k^{\theta_k}}(\Omega) = \frac{1}{2}\delta_{jk} + f_{jk}^{\text{imp}}(\Omega)\overline{S}_{\hat{q}\hat{q}}(\Omega) + \overline{S}_{jk}^{\text{cor}}(\Omega), \tag{S8}$$

where $\frac{1}{2}\delta_{jk}$ is the shot noise homodyne imprecision,

$$\overline{S}_{\hat{q}\hat{q}}(\Omega) = |\chi_{\text{eff}}(\Omega)|^2 \left(2\Gamma_A^{\text{qba}} + 2\Gamma_B^{\text{qba}} + 2\Gamma_m(\bar{n}_{\text{th}} + 1/2) \right) \tag{S9}$$

is the PSD of the mechanical displacement driven by the Brownian force and by the quantum backaction forces $\Gamma_j^{\text{qba}} = g_j^2\kappa_j\left(|u_j(\Omega)|^2 + |v_j(\Omega)|^2\right)$ of the two lasers,

$$f_{jk}^{\text{imp}}(\Omega) = \frac{\sqrt{\Gamma_j^{\text{meas}}\Gamma_k^{\text{meas}}}}{4}\text{Re}\left[e^{-i(\theta_j-\theta_k)}c_{jk}(\Omega) - e^{-i(\theta_j+\theta_k)}\alpha_{jk}(\Omega) \right] \tag{S10}$$

is the transduction function between displacement and detected quadrature and

$$\overline{S}^{\mathrm{cor}}_{jk}(\Omega) = -\frac{\sqrt{\Gamma_j^{\mathrm{meas}}\Gamma_k^{\mathrm{meas}}}}{4}\left(\mathrm{Re}\left[\chi_{\mathrm{eff}}(\Omega)\right]\mathrm{Im}\left[e^{-i(\theta_j+\theta_k)}\alpha_{jk}(\Omega)\right]\right. \\ \left. +\mathrm{Im}\left[\chi_{\mathrm{eff}}(\Omega)\right]\mathrm{Re}\left[e^{-i(\theta_j-\theta_k)}\beta_{jk}(\Omega)\right]\right) \tag{S11}$$

is the correlation between the shot noise and the displacement fluctuations induced by the quantum backaction noise. We also introduced, for the sake of simplicity, the following definitions

$$\alpha_{jk}(\Omega) = \kappa_j\kappa_k\left(\chi_{\mathrm{c},j}(\Omega)\chi_{\mathrm{c},k}(-\Omega) + \chi_{\mathrm{c},k}(\Omega)\chi_{\mathrm{c},j}(-\Omega)\right) = \alpha_{kj}(\Omega), \tag{S12a}$$

$$\beta_{jk}(\Omega) = \kappa_j\kappa_k\left(\chi_{\mathrm{c},j}(\Omega)\chi_{\mathrm{c},k}(\Omega)^* - \chi_{\mathrm{c},j}(-\Omega)\chi_{\mathrm{c},k}(-\Omega)^*\right) = \beta_{kj}(\Omega)^*, \tag{S12b}$$

$$c_{jk}(\Omega) = \kappa_j\kappa_k\left(\chi_{\mathrm{c},j}(\Omega)\chi_{\mathrm{c},k}(\Omega)^* + \chi_{\mathrm{c},j}(-\Omega)\chi_{\mathrm{c},k}(-\Omega)^*\right) = c_{kj}(\Omega)^*, \tag{S12c}$$

$$\Gamma_j^{\mathrm{meas}} = \frac{4g_j^2}{\kappa_j}\,\eta_j\eta_j^{\mathrm{c}}, \tag{S12d}$$

where $\chi_{\mathrm{c},j}(\Omega) = u_j(\Omega) - iv_j(\Omega)$ is the cavity field susceptibility, $\eta_j^{\mathrm{c}} = \kappa_{j,R}/\kappa_j$ the cavity overcoupling, η_j the detection efficiency and Γ_j^{meas} is the measurement rate. The DGCZ criterion for entanglement is based on a pair of EPR observables like $\hat{X}_\pm(\Omega) = \hat{X}_A^{\theta_A}(\Omega) \pm \hat{X}_B^{\theta_B}(\Omega)$ and $\hat{Y}_\pm(\Omega) = \hat{Y}_A^{\theta_A}(\Omega) \pm \hat{Y}_B^{\theta_B}(\Omega)$. The state is entangled if the inseparability $\mathcal{I}(\Omega) < 1$, where

$$\mathcal{I}(\Omega) \equiv \frac{\overline{S}_{\hat{X}_+\hat{X}_+}(\Omega) + \overline{S}_{\hat{Y}_-\hat{Y}_-}(\Omega)}{2} = 1 + f_{q\mathcal{I}}(\Omega)\overline{S}_{\hat{q}\hat{q}}(\Omega) + \mathcal{I}_{\mathrm{cor}}(\Omega), \tag{S13}$$

with

$$f_{q\mathcal{I}}(\Omega) = \frac{1}{4}\mathrm{Re}\left[\Gamma_A^{\mathrm{meas}}c_{AA}(\Omega) + \Gamma_B^{\mathrm{meas}}c_{BB}(\Omega) + 2\sqrt{\Gamma_A^{\mathrm{meas}}\Gamma_B^{\mathrm{meas}}}\alpha_{AB}(\Omega)e^{-\imath 2\Theta}\right], \tag{S14a}$$

$$\mathcal{I}_{\mathrm{cor}}(\Omega) = -\mathrm{Im}[\chi_{\mathrm{eff}}(\Omega)]\mathrm{Re}\left[\frac{\Gamma_A^{\mathrm{meas}}\beta_{AA}(\Omega) + \Gamma_B^{\mathrm{meas}}\beta_{BB}(\Omega)}{4}\right] + \mathrm{Re}[\chi_{\mathrm{eff}}(\Omega)]\mathrm{Im}\left[\frac{\sqrt{\Gamma_A^{\mathrm{meas}}\Gamma_B^{\mathrm{meas}}}\alpha_{AB}(\Omega)e^{-\imath 2\Theta}}{2}\right]. \tag{S14b}$$

and $\Theta = (\theta_A + \theta_B)/2$.

Toy Model

It is useful and instructive to consider the theory presented above in the limits of unresolved-sideband cavity ($\kappa_j \gg \Omega_{\mathrm{m}}, \Delta_j$), resonant drive lasers ($\Delta_j = 0$) and identical measurement rates ($\Gamma_A^{\mathrm{meas}} = \Gamma_B^{\mathrm{meas}} = \Gamma_{\mathrm{meas}}$). These limits, apart from describing clearly the underlying physics, are also well suited for the experiments

described in the main text.

In fact, within these limits, the joint EPR spectrum simplifies to

$$\mathcal{I}(\Omega) \approx 1 + 4\Gamma_{\text{meas}}|\chi_{\text{m}}(\Omega)|^2 \left(2\Gamma_A^{\text{qba}} + 2\Gamma_B^{\text{qba}} + 2\Gamma_m(\bar{n}_{\text{th}} + 1/2)\right)(1 + \cos(2\Theta)) - 4\Gamma_{\text{meas}}\text{Re}[\chi_{\text{m}}(\Omega)]\sin(2\Theta), \quad \text{(S15)}$$

where the quantum backaction rate, in these limits, takes the simple form $\Gamma_j^{\text{qba}} = 4g_j^2/\kappa_j$. Whenever, at any frequency Ω, the value of such spectrum is below the quantum vacuum level 1, the quadrature fluctuations at Ω are entangled. We have already noticed, in the main text, the close analogy between the physics described by Equation () and the case of single continuous field ponderomotive squeezing. A more detailed look at this connection is presented in Supplementary Note 2. Note that the correlations term, which is responsible for the entanglement, always vanishes at the mechanical resonance frequency. One can minimize Equation (S15) over the frequency Ω and the detection angle Θ to show that its lower bound is

$$\mathcal{I}(\Omega) \geq 1 - \frac{\eta_{\text{meas}}}{2}, \quad \text{(S16)}$$

where η_{meas} is the *total* measurement efficiency defined as

$$\eta_{\text{meas}} \equiv \frac{\sum_j \Gamma_j^{\text{meas}}}{\sum_j \Gamma_j^{\text{qba}} + \Gamma_{\text{m}}\left(\bar{n}_{\text{th}} + 1/2\right)} = \frac{2\Gamma_{\text{meas}}}{\Gamma_A^{\text{qba}} + \Gamma_B^{\text{qba}} + \Gamma_{\text{m}}\left(\bar{n}_{\text{th}} + 1/2\right)}. \quad \text{(S17)}$$

Covariance matrix

All the states described above are Gaussian states, due to the quadratic nature of the optomechanical interaction and the assumption of Gaussian white noise at the input. Such a state can be fully described by its covariance matrix $\boldsymbol{\sigma}$, i.e. a symmetric matrix containing the correlations between systems' quadratures: $\sigma_{ij} = \langle x_i x_j + x_j x_i\rangle/2 - \langle x_i\rangle\langle x_j\rangle$, where the state vector $\mathbf{x} = \left(\hat{X}_A, \hat{Y}_A, \hat{X}_B, \hat{Y}_B\right)^{\text{T}}$. Hereby, we consider the covariance matrix of the state right before the detector, i.e. *including* all the optical losses.

This covariance matrix can be described, in block form, by three 2×2 matrices:

$$\boldsymbol{\sigma} = \begin{pmatrix} \alpha & \gamma \\ \gamma^T & \beta \end{pmatrix}. \quad \text{(S18)}$$

Two of these submatrices, α and β, describe the individual subsystems while the third, γ, describes the correlations between these subsystems,

A sufficient criterion for such state to be entangled is that $2\tilde{\nu}_- < 1$, where $\tilde{\nu}_-$ is the lowest symplectic eigenvalue of the partial transposed covariance matrix[2]. Given the matrix in Equation (S18), the minimum symplectic eigenvalues $\tilde{\nu}_-$ is:

$$\tilde{\nu}_- = \sqrt{\frac{\Delta(\boldsymbol{\sigma}) - \sqrt{\Delta(\boldsymbol{\sigma})^2 - 4\mathrm{Det}\boldsymbol{\sigma}}}{2}}, \tag{S19}$$

where $\Delta(\boldsymbol{\sigma}) = \mathrm{Det}\alpha + \mathrm{Det}\beta - 2\mathrm{Det}\gamma$. The physical meaning of $\tilde{\nu}_-$ can be understood as[3] $2\tilde{\nu}_- = \min \mathcal{I}(\Omega)$, where the inseparability is minimized over all local linear unitary Bogolioubov operations, such as rotations and squeezing. Thus, $\tilde{\nu}_-$ sets a lower bound of the DGCZ inseparability at arbitrary quadratures. The minimum symplectic eigenvalue of the partial transposed covariance matrix is related to well-known entanglement measure logarithmic negativity E_N as $E_N = \max[0, -\ln 2\tilde{\nu}_-]$.

For the toy model defined above, the covariance matrix can be written as:

$$\mathbf{V} = \begin{pmatrix} \frac{1}{2} & 2\Gamma_{\mathrm{meas}}\mathrm{Re}[\chi_m(\Omega)] & 0 & 2\Gamma_{\mathrm{meas}}\mathrm{Re}[\chi_m(\Omega)] \\ 2\Gamma_{\mathrm{meas}}\mathrm{Re}[\chi_m(\Omega)] & \frac{1}{2} + 8\Gamma_{\mathrm{meas}}|\chi_m(\Omega)|^2\Gamma_{\mathrm{dec}} & 2\Gamma_{\mathrm{meas}}\mathrm{Re}[\chi_m(\Omega)] & 8\Gamma_{\mathrm{meas}}|\chi_m(\Omega)|^2\Gamma_{\mathrm{dec}} \\ 0 & 2\Gamma_{\mathrm{meas}}\mathrm{Re}[\chi_m(\Omega)] & \frac{1}{2} & 2\Gamma_{\mathrm{meas}}\mathrm{Re}[\chi_m(\Omega)] \\ 2\Gamma_{\mathrm{meas}}\mathrm{Re}[\chi_m(\Omega)] & 8\Gamma_{\mathrm{meas}}|\chi_m(\Omega)|^2\Gamma_{\mathrm{dec}} & 2\Gamma_{\mathrm{meas}}\mathrm{Re}[\chi_m(\Omega)] & \frac{1}{2} + 8\Gamma_{\mathrm{meas}}|\chi_m(\Omega)|^2\Gamma_{\mathrm{dec}} \end{pmatrix}, \tag{S20}$$

where $\Gamma_{\mathrm{dec}} = \Gamma_A^{\mathrm{qba}} + \Gamma_B^{\mathrm{qba}} + \Gamma_{\mathrm{m}}(\bar{n}_{\mathrm{th}} + 1/2)$. The minimum symplectic eigenvalue can be promptly calculated to be

$$2\tilde{\nu}_-(\Omega) = \sqrt{1 + 16\Gamma_{\mathrm{meas}}|\chi_{\mathrm{m}}(\Omega)|^2\Gamma_{\mathrm{dec}}\left(1 - \sqrt{1 + \frac{\mathrm{Re}[\chi_{\mathrm{m}}(\Omega)]^2}{4|\chi_{\mathrm{m}}(\Omega)|^4\Gamma_{\mathrm{dec}}^2}}\right)}. \tag{S21}$$

If we focus on the vicinity of mechanical frequency ($|\delta_{\mathrm{m}}| = |\Omega_{\mathrm{m}} - \Omega| \ll \Omega_{\mathrm{m}}$), the ratio in the inner square root $\mathrm{Re}[\chi_{\mathrm{m}}(\Omega)]^2/4|\chi_{\mathrm{m}}(\Omega)|^4\Gamma_{\mathrm{dec}}^2 \approx \delta_{\mathrm{m}}^2/\Gamma_{\mathrm{dec}}^2$. In the limit of $\Gamma_{\mathrm{dec}} \gg \delta_{\mathrm{m}}$, we can take the approximation $\sqrt{1 + \delta_{\mathrm{m}}^2/\Gamma_{\mathrm{dec}}^2} \approx 1 + \delta_{\mathrm{m}}^2/(2\Gamma_{\mathrm{dec}}^2)$. At the same time, in the limit of $\delta_{\mathrm{m}} \gg \Gamma_{\mathrm{m}}$, $4|\chi_{\mathrm{m}}(\Omega)|^2 \approx 1/\delta_{\mathrm{m}}^2$. Then the expression can be simplified to:

$$2\tilde{\nu}_-(\Omega) \approx \sqrt{1 - \frac{2\Gamma_{\mathrm{meas}}}{\Gamma_{\mathrm{dec}}}} = \sqrt{1 - \eta_{\mathrm{meas}}}. \tag{S22}$$

Thus, in the limit of very strong measurement strength $\eta_{\mathrm{meas}} \to 1$ one can achieve arbitrarily strong entanglement for optical modes at frequency δ_{m}, where $\Gamma_{\mathrm{m}} \ll \delta_{\mathrm{m}} \ll \Gamma_{\mathrm{dec}}$. Though the last expression is frequency independent, deviation from the limits results in the frequency dependence feature of Figure 4 in main text.

Supplementary Note 2: Entanglement as Joint Quadrature Squeezing

Here, we comment further on the toy model presented in Supplementary Note 1, to highlight the form of correlations generated in this system. First, recall that, in the toy model, the dynamics of the optical modes are given by:

$$\hat{X}_j^{\text{out}} = -\hat{X}_j^{\text{in}}, \tag{S23a}$$

$$\hat{Y}_j^{\text{out}} = -\hat{Y}_j^{\text{in}} - 2\sqrt{\Gamma_{\text{qba}}}\chi_{\text{m}}(t) * \left(\sqrt{2\Gamma_{\text{m}}}\hat{P}_{\text{in}} + \sqrt{4\Gamma_{\text{qba}}}\hat{X}_+^{\text{in}}\right), \tag{S23b}$$

where we also assume, for the sake of clarity, equal cavity linewidths $\kappa = \kappa_A = \kappa_B$

The links between the different optical and mechanical quadratures are illustrated in Supplementary Figure 1(a). As mentioned in the main text, there is self-squeezing as well as cross-correlations, both generated by amplitude fluctuations driving motion, which then drives phase fluctuations. If we move to the basis of the joint sum and difference quadratures, $\hat{X}_\pm = \hat{X}_A \pm \hat{X}_B$ and $\hat{Y}_\pm = \hat{Y}_A \pm \hat{Y}_B$ (applied to both input and output fields), we can rewrite the dynamics as follows:

$$\hat{X}_+^{\text{out}} = -\hat{X}_+^{\text{in}}, \tag{S24a}$$

$$\hat{Y}_+^{\text{out}} = -\hat{Y}_+^{\text{in}} - 4\sqrt{\Gamma_{\text{qba}}}\chi_{\text{m}}(t) * \left(\sqrt{2\Gamma_{\text{m}}}\hat{P}_{\text{in}} + \sqrt{4\Gamma_{\text{qba}}}\hat{X}_+^{\text{in}}\right), \tag{S24b}$$

$$\hat{X}_-^{\text{out}} = -\hat{X}_-^{\text{in}}, \tag{S24c}$$

$$\hat{Y}_-^{\text{out}} = -\hat{Y}_-^{\text{in}}. \tag{S24d}$$

Note that the difference mode becomes completely de-coupled from the optomechanical dynamics, simply remaining in its initial vacuum state. Meanwhile, the sum mode undergoes the usual optomechanical inter-

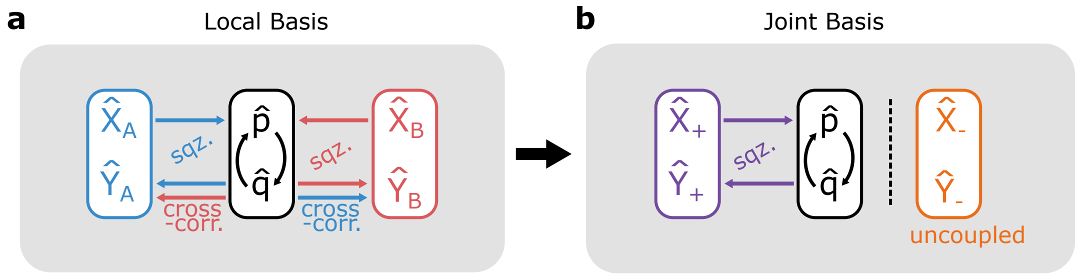

Supplementary Figure 1: **Dynamics in the Joint Mode Basis. a**, Schematic illustration of the couplings between the optical quadratures $(\hat{X}_A, \hat{Y}_A, \hat{X}_B, \hat{Y}_B)$ and mechanical position/momentum (q, p). Each laser generates self-squeezing as well as cross-correlations. **b**, Schematic illustration of the couplings after moving to the (non-local) joint basis $(\hat{X}_\pm = \hat{X}_A \pm \hat{X}_B, \hat{Y}_\pm = \hat{Y}_A \pm \hat{Y}_B)$. Here, there is only a self-squeezing of the sum-mode, while the difference-mode remains uncoupled from all system dynamics.

action, generating self-squeezing through the mechanical motion. This squeezing is twice as strong as the self-squeezing of either individual laser.

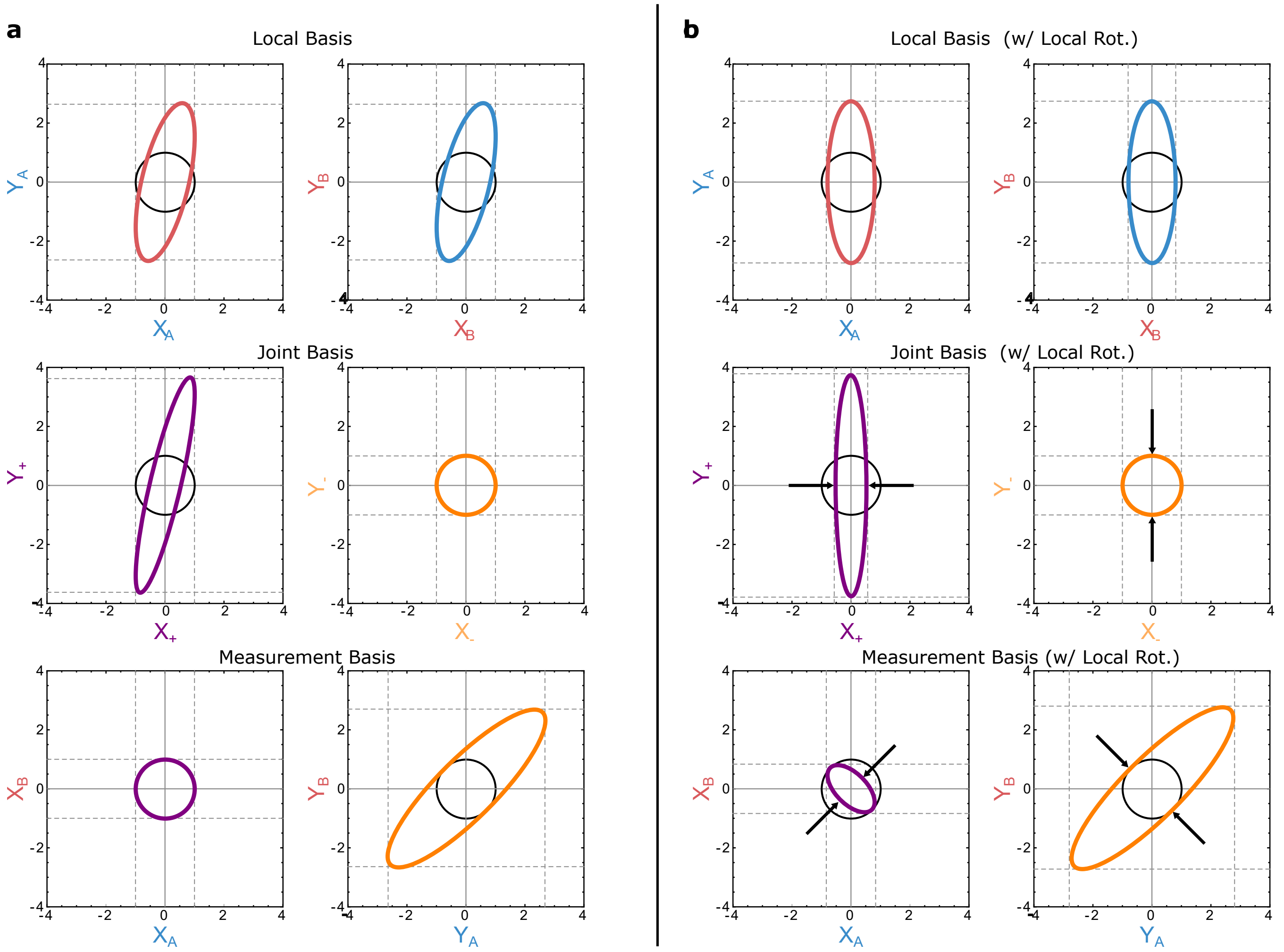

Supplementary Figure 2: **Entanglement in Phase Space. a**, Different phase space portraits of the system correlations (i.e. different cuts of the 4-dimensional covariance ellipsoid). In all subplots, the black circle indicates vacuum noise, and the dashed lines indicate the marginal variances along each axis. The top row is in the (local) cavity quadrature basis, where each system simply exhibits self-squeezing. The middle row moves to the joint bases, where we see that the difference mode remain in vacuum, while the sum-mode displays self-squeezing (with a non-zero squeezing angle). We note that the squeezing of this joint quadrature is stronger than the sub-system self-squeezing. The bottom row corresponds to the ellipses we are able to directly measure in our system (i.e. simultaneous $\{\hat{X}_A, \hat{X}_B\}$ or $\{\hat{Y}_A, \hat{Y}_B\}$). **b**, Similar noise ellipses as in **a**, but with a local rotation of the A,B subsystems (i.e. $\theta_A = \theta_B \neq 0$). As a result, the joint-basis ellipses (middle row) are aligned such that we now see $V(X_+) + V(Y_-)$ will violate the DGCZ criterion, as indicated by the black arrows. In our measurements, these variances are extracted from the diagonal/anti-diagonal variances (bottom row). We note that the bottom row closely matches the experimental noise ellipses of Figure 2 in the main text.

Supplementary Figure 2 shows covariance ellipses based on this model, illustrating how the correlations appear in either basis. (Note that in the figure and discussion below, we refer only to output modes, but drop the ^{out} superscript for convenience.) We note that the local-basis ellipses are insufficient to characterize the system, since there are unseen correlations (Y_A and Y_B, for example). On the other hand, since the joint quadrature bases are decoupled, these covariance ellipses completely describe all significant system correlations.

To link these covariance ellipses to entanglement, recall that the DGCZ criterion requires that $\left(V(X_+) + V(Y_-)\right)/2 < 1$. For the above quadrature definitions, this is not yet true, since the squeezing of the sum mode occurs for some non-zero squeezing angle (see Supplementary Figure 2(a)). However, by applying a local rotation to each laser (i.e. selecting $\theta_A = \theta_B \neq 0$), we can align this sum-mode-squeezing with the basis, such that we find $V(X_+) < 1$, while $V(Y_-)$ remains at the vacuum level. Thus, we see how self-squeezing of this non-local mode establishes the entanglement of the two fields.

Supplementary Note 3: Output spectral modes and homodyne detection

So far, we have been discussing about bipartite entanglement, i.e. entanglement between two modes. In general, the dynamics described before involves four *spectral* modes. We show now that, with some assumptions on the underlying quantum state and homodyne measurements, this quadripartite state can effectively be reduced to a simpler bipartite state[4].

A single, continuous field $\hat{a}(t) = \int \frac{d\Omega}{2\pi} e^{-\imath\Omega t}\hat{a}_\Omega$ can be seen as a highly multimode system, containing an ensemble of spectral modes $\hat{a}_\Omega$. The bandwidth of this ensemble is defined by the details of the system which generates the field. The optomechanical interaction in Equation (S2) simultaneously affects the *sideband* modes (a pair of spectral modes symmetric around the carrier frequency) $\hat{a}_{\Omega_L \pm \Omega_\mathrm{m}}$. To keep the notation simple and concise, we drop, in the subscripts, the carrier frequency Ω_L in the following.

In our case, a multimode optomechanical system is driven by two different fields, $\hat{a}(t)$ and $\hat{b}(t)$, which interact with the same mechanical mode. Thus, the interaction involves four spectral modes, i.e. two pairs of sideband modes $\hat{a}_{\pm\Omega_\mathrm{m}}$ and $\hat{b}_{\pm\Omega_\mathrm{m}}$, each around its own carrier frequency, as shown in Supplementary Figure 3.

Subsequently, each field is measured via spectral homodyne detection, which corresponds to a Fourier analysis of the homodyne photocurrent, in order to resolve individual spectral modes. This results in a mixing of the upper and lower sideband at the analysis frequency Ω. For a single field, given the photocurrent

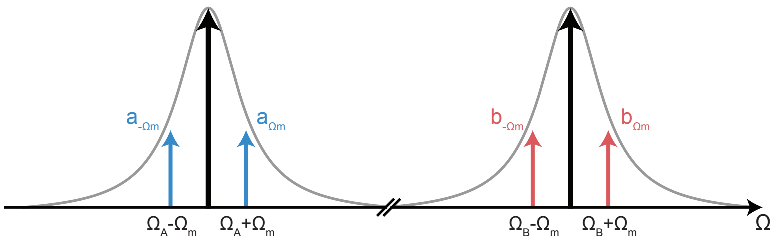

Supplementary Figure 3: **Output spectral modes involved.** Each laser field drives, resonantly, an optical cavity mode and interact with a common mechanical mode of frequency $\Omega_{\rm m}$. As a result, the output propagating fields posses non-trivial sideband modes at the mechanical frequency $\Omega_{\rm m}$. Thus, their dynamics is determined by a quadripartite state.

$\hat{i}(t) = e^{-\imath\theta}\hat{a}(t) + e^{\imath\theta}\hat{a}(t)^{\dagger}$, its Fourier component at frequency Ω is

$$\hat{i}_{\Omega} = \frac{e^{-\imath\theta}\hat{a}_{\Omega} + e^{\imath\theta}(\hat{a}_{-\Omega})^{\dagger}}{\sqrt{2}} = \cos(\theta)\,\frac{\hat{X}_{\rm s} + \imath\hat{Y}_{\rm a}}{\sqrt{2}} + \sin(\theta)\,\frac{\hat{Y}_{\rm s} - \imath\hat{X}_{\rm a}}{\sqrt{2}}, \tag{S25}$$

where $\hat{X}_{\rm s(a)}$, $\hat{Y}_{\rm s(a)}$ are the quadratures of the symmetric (antisymmetric) modes, defined as $\hat{a}_{\rm s(a)} = (\hat{a}_{\Omega} \pm \hat{a}_{-\Omega})/\sqrt{2}$. Equation (S25) shows us that spectral homodyne detection is truly a two-mode measurement, where the two modes involved are the upper and lower sidebands $\hat{a}_{\pm\Omega}$ or, equivalently, the symmetric and antisymmetric modes. When considering the case with two fields, the combined homodyne measurements directly sample the quadripartite state formed by the spectral modes $(\hat{a}_{\Omega}, \hat{a}_{-\Omega}, \hat{b}_{\Omega}, \hat{b}_{-\Omega})$.

Generally speaking, such state cannot be completed reconstructed from homodyne detection, as this measurement is "blind" to several correlation terms[4]. However, an important special case (valid also for our system) is played by stationary states with no asymmetry between the energy of sideband modes. Within these assumptions, the quadripartite state simplifies to a bipartite one formed only by the symmetric mode of the two fields. We also notice that, in this case, the antisymmetric mode possesses the same quantum statistics as the symmetric one, apart from a local (then irrelevant for entanglement) rotation in phase space.

This would suggest than a single-mode measurement description for spectral homodyne detection is possible. However, the measured quadratures, e.g. Equation (S7), are not proper single-mode quadrature operators (in fact, orthogonal quadratures commute, i.e. $[\hat{X}_{\Omega}, \hat{Y}_{\Omega}] = 0$) but rather semiclassical ones. Nevertheless, for stationary quantum states, they behave as effective single-mode quadrature operators as far as second-order moments are concerned, when the right prescription to calculate noise power is used. Then, from their statistics, one can reconstruct the covariance matrix for the symmetric modes. It is the entanglement of these symmetric modes, belonging to the two fields, that we observe and report in the main text[4].

We also stress that this represents a different situation compared to red-blue sideband entanglement generated by a resolved-sideband optomechanical system[5]. In our case, the correlations of interest are present

in the electronic signal at frequency $\Omega_{\rm m}/(2\pi) \sim \mathcal{O}(1\,{\rm MHz})$, where technical noise can be suppressed and quantum limited detection achieved. In the resolved sideband case, instead, the two entangled modes are single spectral modes and not sideband modes. The correlations, in the homodyne detection signal, are now present at zero frequency, where technical noise obstructs quantum-limited measurements. This can be alleviated by employing an heterodyne detector, which, however, would limit the detection efficiency to 50%.

Supplimentary Note 4: Mode Decomposition by Fourier Transform

In the main text, we introduce temporal modes (defined in Equation (3)), obtained by filtering the optical quadratures with a kernel, $h(t)$. In the data analysis of Figures 2 and 3, we construct such modes using an exponential kernel (in practice, applying a digital, 4th order butterworth filter to the mixed-down signal). In Figure 4, we wish to analyze the frequency-dependent statistics of such modes. For computational speed, we accomplish this by calculating the FFT of the photocurrents, which corresponds to using a rectangular/boxcar kernel in Equation (3). While the profile of this FFT-defined mode will differ slightly from the modes of Figs. 2 and 3, we note here that the differences are not significant for our analysis. Supplementary Figure 4 compares, for example, the frequency dependence of $\tilde{\nu}_{-}$, for modes calculated by Fourier transform and by exponential kernel.

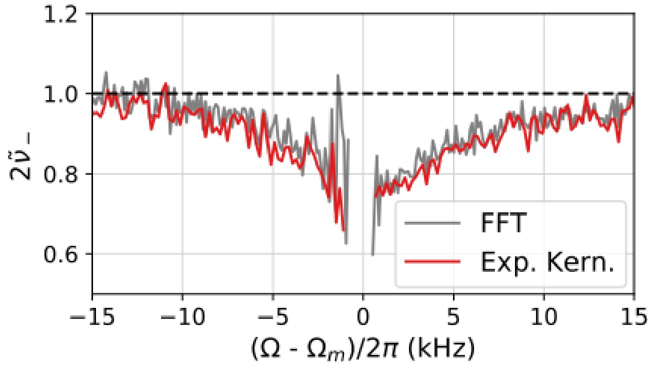

Supplementary Figure 4: **Comparison of temporal mode definitions.**

Supplementary Note 5: Fitting of power spectral densities

Here we describe the analysis procedure for fitting the spectra shown in Figure 4 of the main text (the frequency-dependent DGCZ measurements). Recall that each measurement run (i.e. each joint basis angle

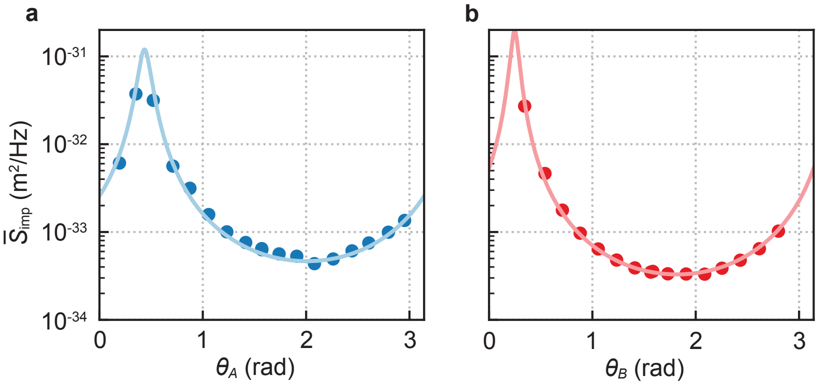

Supplementary Figure 5: **Imprecision for different quadratures. a**, **b**, Displacement imprecision for laser A and B, respectively, as a function of their homodyne angle. The light lines are fits.

Θ) involves three sequential measurements, each of which consist of two simultaneously acquired photocurrent streams. These three steps measure (1) shot noise (by blocking cavity outputs), (2) $\{\hat{X}_A^{\theta_A}, \hat{X}_B^{\theta_B}\}$, and (3) $\{\hat{Y}_A^{\theta_A}, \hat{Y}_B^{\theta_B}\}$ (by advancing the homodyne angles from step (2) by $\pi/2$). We start by fitting the detection efficiency of each system. Following a standard technique[6], we can use a phase modulation tone in the spectra to calibrate the shot noise background into displacement units. We fit these displacement spectra (at various homodyne angles) to $\overline{S}_j^{\mathrm{imp}}(\theta_j) = 2x_{\mathrm{ZPF}}^2/f_{jj}^{\mathrm{imp}}(\overline{\Omega})$, where $f_{jj}^{\mathrm{imp}}(\overline{\Omega})$ is defined in Equation (S10) and $\overline{\Omega}$ is the fixed Fourier frequency at which we measure the background value (see Supplementary Figure 5). From these fits we get $\eta_A = 60\%$ and $\eta_B = 77\%$. (Note that in this and future fits, the exact values of θ_A and θ_B are extracted from DC values of the balanced photocurrents.)

We then move to analyze and fit the photocurrent spectra from from steps (2) and (3). Each measurement run yields 6 different spectra: $\overline{S}_{\hat{X}_A^{\theta_A}\hat{X}_A^{\theta_A}}, \overline{S}_{\hat{X}_B^{\theta_B}\hat{X}_B^{\theta_B}}, \mathrm{Re}\left(\overline{S}_{\hat{X}_A^{\theta_A}\hat{X}_B^{\theta_B}}\right)$ and the analogous for $\hat{Y}_j^{\theta_j}$. We normalize them to the shot noise level acquired in step (1). We fit all 6 spectra simultaneously to Equation (S8) with the appropriate choice of angles and labels j, k. The only free parameters in this fit are the coupling g_j and the detuning Δ_j, in order to account for slow drift during the measurements. An example of such fitted spectra is given in Supplementary Figure 6. Finally, we show in Supplementary Figure 7 the fitted parameters for all the measurement runs. Their mean values are used to plot theory lines in Figure 2 and Figure 3 of the main text.

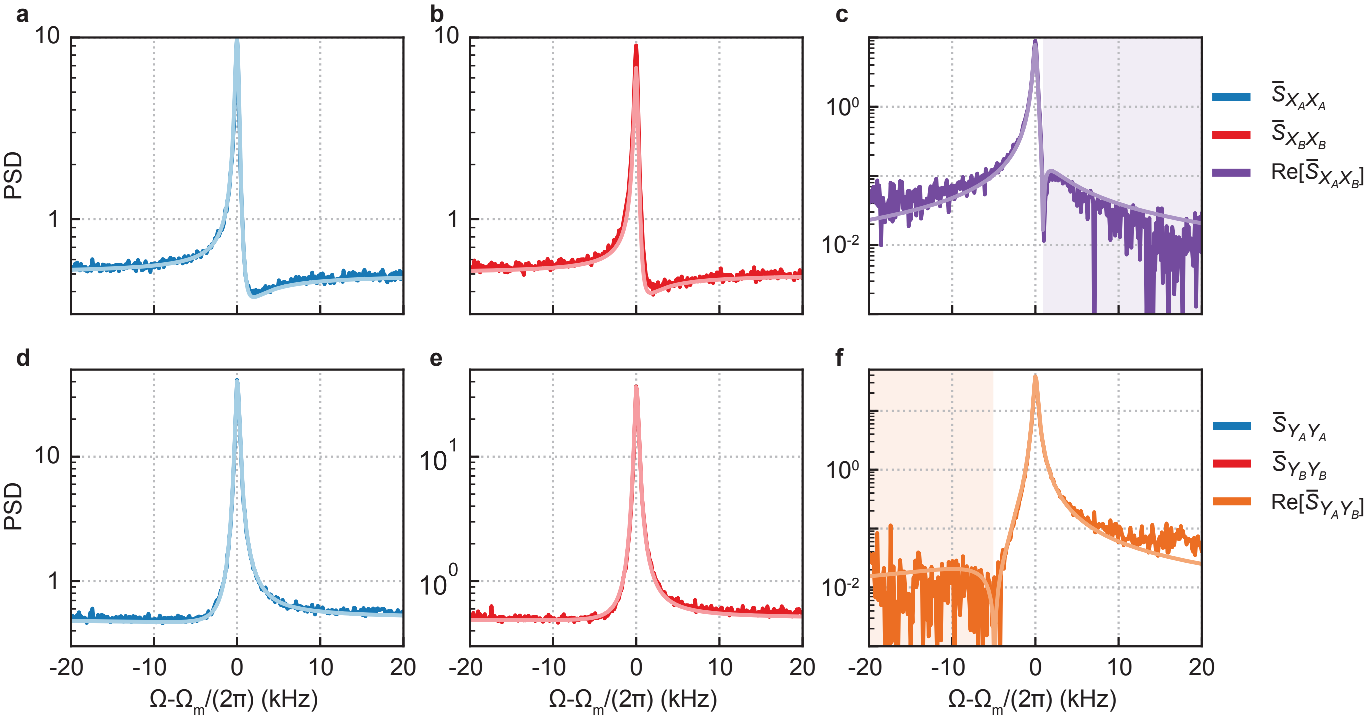

Supplementary Figure 6: **Power spectral density from a measurement run. a**, **b**, **c**, PSDs for the $\hat{X}$ quadratures of the two lasers ($\Theta \approx 0$). We show the absolute value of the cross-spectrum in **c** for the sake of visualization and note that the shaded area is actually negative. Light-coloured lines are the result of a simultaneous fit of all 6 spectra. **d**, **e**, **f**, PSDs for the $\hat{Y}$ quadratures of the two lasers.

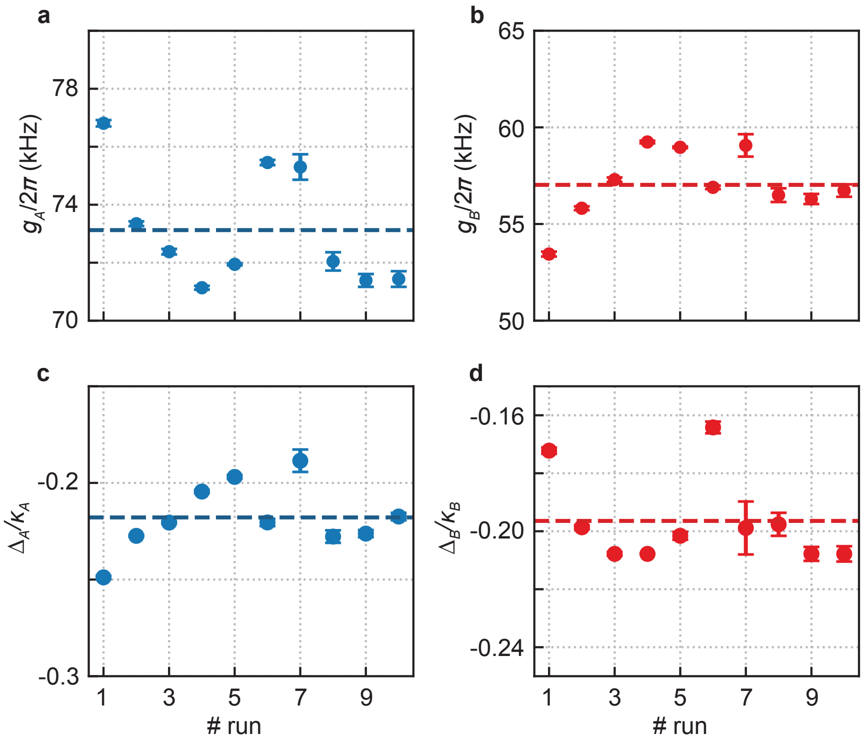

Supplementary Figure 7: **Fitted parameters for all measurement runs. a**, **b**, Fitted optomechanical coupling g for laser A and B, respectively. **c**, **d**, Fitted detuning Δ for laser A and B, respectively, in units of the respective cavity linewidth. The dashed lines are mean values. Error bars represents a single standard deviation coming from the fits.

Supplementary Note 6: Calibration of balanced homodyne detector

In general, the main results of the manuscript depend only on comparing measured spectra/variances to a shot noise reference level. To measure this reference level, we block the signal, so only the equally-distributed local oscillator reaches the balanced homodyne detector (BHD). The conditions of this measurement differ from a generic quadrature measurement, where the BHD is locked at angle θ and the power on each diode can differ, due to interference between the LO and the coherent signal from the experiment. In principle, this can lead to small systematic effects related to photodiode gain differences and classical laser noise. These effects can be characterized by shot noise measurements in which the LO power is unbalanced[7], as we derive below. (We note already that no significant systematic artifacts are present in our data, but we present this characterization for completeness.)

We consider optical fields at the photodiodes of a balanced homodyne detector, with real local oscillator field α_{LO}, real signal field α_{s}, and classical laser amplitude noise $n(t)$ (see Supplementary Figure 8**a**). The local oscillator power is imbalanced by a (relative) amount δ. The field at diode $\pm$, where $+$ or $-$ refers to the different photodiodes, is given by:

$$\frac{\alpha_{\mathrm{LO}}}{\sqrt{2}}\sqrt{1\pm\delta}(1+n(t))e^{-i\theta} \pm \frac{\alpha_{\mathrm{s}}}{\sqrt{2}}(1+n(t)) + \hat{a}_{\pm,\mathrm{vac}}, \tag{S26}$$

where θ is the relative phase between LO and signal, $\hat{a}_{\pm,\mathrm{vac}}$ is the vacuum noise, and the $\pm$ before α_{s} term results from reflection of field from the final beam splitter. As the variance of vacuum $\hat{a}_{\pm,\mathrm{vac}}$ is invariant under rotation, the photocurrent spectrum is completely determined by the amplitude of the classical terms. In a balanced configuration ($\delta = 0$), this amplitude is given by

$$(1+n(t))\sqrt{\frac{\alpha_{\mathrm{LO}}^2}{2}+\frac{\alpha_{\mathrm{s}}^2}{2}\pm\alpha_{\mathrm{LO}}\alpha_{\mathrm{s}}\cos\theta} \approx (1+n(t))\sqrt{\frac{\alpha_{\mathrm{LO}}^2}{2}\pm\alpha_{\mathrm{LO}}\alpha_{\mathrm{s}}\cos\theta}, \tag{S27}$$

where we use the assumption that $\alpha_{\mathrm{LO}}^2 \gg \alpha_{\mathrm{s}}^2$. Alternatively, if we block the signal and consider an unbalanced ($\delta \neq 0$) detector, the amplitude is given by:

$$(1+n(t))\sqrt{\frac{\alpha_{\mathrm{LO}}^2}{2}(1\pm\delta)}. \tag{S28}$$

Comparing these two cases, we find that Equations (S27) and (S28) are equivalent when $\delta = 2\alpha_{\mathrm{s}}/\alpha_{\mathrm{LO}}\cos\theta$. Thus, all systematic artifacts caused by measuring $\theta \neq \pi/2$, can be completely characterized by blocking the signal ($\alpha_{\mathrm{s}} = 0$) and unbalancing the LO. In practice, we don't measure δ but the amplified DC component of the photocurrent, $V_{\mathrm{DC}} = \alpha_{\mathrm{LO}}^2/2\,(g_+ - g_- + (g_+ + g_-)\delta)$. The power spectral density of the amplified differential photocurrent, V is

$$\overline{S}_{VV}(\Omega) = \underbrace{g_+ g_- \alpha_{\mathrm{LO}}^2 + (g_+ - g_-)\,V_{\mathrm{DC}}}_{\text{shot noise}} + \underbrace{4\overline{S}_{\mathrm{nn}}(\Omega)V_{\mathrm{DC}}^2}_{\text{classical noise}}, \tag{S29}$$

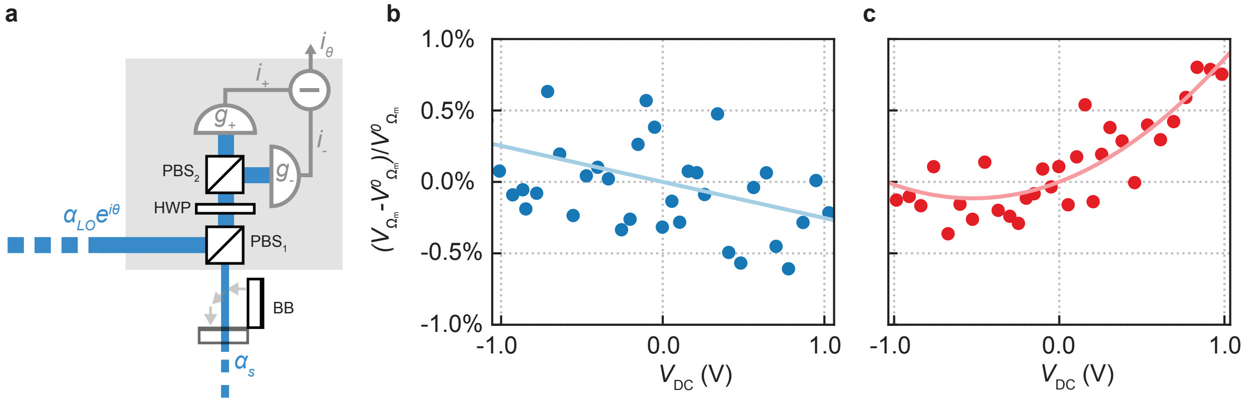

Supplementary Figure 8: **Systematic errors in balanced homodyne detectors. a**, A local oscillator with coherent amplitude $\alpha_{\mathrm{LO}}e^{\imath\theta}$ is combined with an orthogonally linearly polarized field α_s on a polarizing beam splitter (PBS). A half-wave plate (HWP) rotates the polarization by $45°$ (in a balanced configuration) and a final PBS splits the mixed fields. Two photodiodes (labeled $+$ and $-$) with overall gain $g_\pm$ detect the fields. The resulting photocurrent $i_\pm$ are then subtracted and the differential photocurrent i is given as output. In the experiment, two such balanced homodyne detectors are used (one for each laser). To calibrate the detector, the signal field can be blocked by rotating the beam block (BB) and the local oscillator power can be unbalanced by rotating the HWP. **b**, **c**, Difference in measured variance at around Ω_{m}, relative to the variance when the detector is balanced, as a function of the DC component of the differential photocurrent (here in terms of voltage) for, respectively, laser A and laser B. Every V_{DC} corresponds to a rotation of the HWP. The light blue (red) line is fit to a linear (quadratic) function.

where $g_\pm$ are the photodiode gains and $\overline{S}_{\mathrm{nn}}(\Omega)$ is the power spectral density of the amplitude noise $n(t)$. We vary V_{DC} by changing the splitting ratio δ and measure the average spectral noise $V_{\Omega_{\mathrm{m}}}$ at around Ω_{m}. In Supplementary Figure 8**b** and **c** we show, respectively, the relative variance $(V_{\Omega_{\mathrm{m}}} - V^0_{\Omega_{\mathrm{m}}})/V^0_{\Omega_{\mathrm{m}}}$ for the BHD of laser A and B, where $V^0_{\Omega_{\mathrm{m}}}$ is the variance at $V_{\mathrm{DC}} = 0$, which approximately corresponds to a balanced detector for small gain difference. The variance $V^0_{\Omega_{\mathrm{m}}}$ is also the one used as a reference shot noise for the main results in the main text. The largest deviation we observe ($< 1\%$) is much less then any DGCZ violations reported here and, thus is insignificant. Nevertheless, we consider such systematics for the best inseparability value reported in the main text. For this measurement, we operate the detectors both at the minimum of the fringe interference (i.e. $V_{\mathrm{DC}} \approx -1$ V) and at the center ($V_{\mathrm{DC}} \approx 0$ V). We see that an error of $\sim 0.3\%$ arises when measuring the amplitude quadratures of laser A.

Supplementary Note 7: System parameters

In Supplementary Table 1 we collect the main symbols and parameters which have been used in this work.

Symbol	Definition	Name	Value mode A	Value mode B
Ω_m		Mechanical resonance frequency	$2\pi\times 1.139$ MHz	
Γ_m		Mechanical linewidth	$2\pi\times 1.1$ mHz	
Q	$\Omega_\mathrm{m}/\Gamma_\mathrm{m}$	Mechanical quality factor	1.03×10^9	
T		Mechanical bath temperature	10 K	
$\bar{n}_\mathrm{th}$	$(e^{\hbar\Omega_\mathrm{m}/\kappa_B T}-1)^{-1}$	Thermal phonon occupation	1.8×10^5	
m_eff		Effective mass	2.3 ng	
x_zpf	$\sqrt{\frac{\hbar}{2m_\mathrm{eff}\Omega_\mathrm{m}}}$	Zero point fluctuations	1.8 fm	
λ_j		Laser wavelength	796.154 nm	796.750 nm
g_j		Field-enhanced optomechanical coupling	$2\pi\times 67.0$ kHz	$2\pi\times 53.1$ kHz
κ_j		Cavity linewidth	$2\pi\times 13.3$ MHz	$2\pi\times 12.6$ MHz
Δ_j		Laser-cavity detuning	$-0.22\,\kappa_A$	$-0.20\,\kappa_B$
η_j^c		Cavity overcoupling	95%	95%
η_j		Detection efficiency	60%	77%
Γ_j^meas	$\eta_j\eta_j^\mathrm{c}\,4g_j^2/\kappa_j$	Measurement rate	$2\pi\times$ 0.77kHz	$2\pi\times$ 0.65kHz
Γ_j^qba	$4g_j^2/\kappa_j$	Measurement-induced quantum backaction rate	$2\pi\times$ 1.35kHz	$2\pi\times$ 0.89kHz
γ	$\Gamma_\mathrm{m}\,(\bar{n}_\mathrm{th}+1/2)$	Thermal decoherence rate	$2\pi\times 202$ Hz	
η_meas	$\sum_j \frac{\Gamma_j^\mathrm{meas}}{\Gamma_j^\mathrm{qba}+\gamma}$	Total measurement efficiency	58 %	
Γ_dec	$\Gamma_A^\mathrm{qba}+\Gamma_B^\mathrm{qba}+\gamma$	Total decoherence rate	$2\pi\times 2.44$ kHz	
θ_j		Homodyne detection angle		
Θ	$(\theta_A+\theta_B)/2$	joint homodynes angle		
$\hat{X}_j^{\theta_j}$		Quadrature of optical mode		
$\hat{X}_\pm$	$\hat{X}_A^{\theta_A}\pm\hat{X}_B^{\theta_B}$	EPR amplitude quadratures		
$\hat{Y}_\pm$	$\hat{Y}_A^{\theta_A}\pm\hat{Y}_B^{\theta_B}$	EPR phase quadratures		
ν_-		Minimum symplectic eigenvalue		
$S_{\hat{A}\hat{B}}(\Omega)$	$\int dt e^{\imath\Omega t}\langle\hat{A}(t)\hat{B}(0)\rangle$	Power spectral density		
$\overline{S}_{\hat{A}\hat{B}}(\Omega)$	$\frac{S_{\hat{A}\hat{B}}(\Omega)+S_{\hat{B}\hat{A}}(-\Omega)}{2}$	Symmetrized PSD		

Supplementary Table 1: Parameters and definitions.

Supplementary References

[1] Giovannetti, V. & Vitali, D., Phase-noise measurement in a cavity with a movable mirror undergoing quantum Brownian motion. *Physical Review A* **63**, 023812 (2001).

[2] Adesso, G., Serafini, A. & Illuminati, F., Extremal entanglement and mixedness in continuous variable systems. *Physical Review A* **70**, 022318 (2004).

[3] Zippilli, S., Di Giuseppe, G. & Vitali, D., Entanglement and squeezing of continuous-wave stationary light. *New Journal of Physics* **17**, 4 043025 (2015).

[4] Barbosa, F. A. S., Coelho, A. S., Cassemiro, K. N., Nussenzveig, P., Villar, A. S. & Martinelli, M., Quantum state reconstruction of spectral modes: Homodyne and resonator detection schemes. *Physical Review A* **88**, 052113 (2013).

[5] Barzanjeh, S., Redchenko, E. S., Peruzzo, M., Wulf, M., Lewis, D. P. and Fink, J. M. Stationary entangled radiation from micromechanical motion. *Nature* **570**, 480–483 (2019).

[6] Gorodetsky, M. L., Schliesser, A., Anetsberger, G., Deleglise, S. & Kippenberg, T. J., Determination of the vacuum optomechanical coupling rate using frequency noise calibration. *Optical Express* **18**, 22 23236 (2010).

[7] Safavi-Naeini, A. H., Gröblacher, S., Hill, J. T., Chan, J., Aspelmeyer, M. & Painter, O., Squeezed light from a silicon micromechanical resonator. *Nature* **500**, 185–189 (2013).
